# Proteomic and Amino Acid Dynamics During Jujube Blackening: Structural Transformations and Maillard Reaction Linkages

**DOI:** 10.1002/fsn3.70644

**Published:** 2025-07-25

**Authors:** Weihao Kong, Xin Sun, Xin Zhang, Yuxiao Wang, Yifei Zhao, Lin Gao, Lingwei Bu, Rentang Zhang

**Affiliations:** ^1^ College of Food Science and Engineering Shandong Agricultural University Tai'an Shandong China; ^2^ Laoling Healthy Food Industry Technology Research Institute Dezhou China

**Keywords:** amino acid, blackened jujube, Maillard reaction, protein, structural characterization

## Abstract

In this study, the structural characteristics of proteins and the amino acid composition during the jujube blackening process were investigated, with a particular focus on their relationship to the Maillard reaction. The reaction induced disruption of protein covalent bonds and altered protein aggregation states, with the most significant structural changes observed at 78 h. Blackened jujube exhibited elevated levels of pyruvate dehydrogenase and a higher content of hydrophobic amino acids compared to hydrophilic ones, both of which facilitated the progression of the Maillard reaction. The contents of total and free amino acids underwent marked changes throughout the blackening process, with substantial reductions in most amino acids, except for a few. After darkening, valine was identified as the most abundant essential amino acid, accounting for 68.69% of the total amino acid content, while cysteine was the most prevalent nonessential amino acid at 13.38%. The protein with the highest abundance had a molecular weight of approximately 60 kDa. Overall, this study elucidates the effects of the Maillard reaction on jujube proteins and amino acids and provides a theoretical foundation for understanding the formation mechanism of blackened jujube.

## Graphical abstract

This study characterized the protein structure and amino acid content changes in jujube during the blackening process, which is likely linked to the Maillard reaction. The reaction caused protein covalent bond disruption, aggregation, and significant structural changes after 6 h, with blackened jujube showing higher levels of pyruvate dehydrogenase and hydrophobic amino acids. Essential amino acids, particularly valine (68.69%), and non‐essential amino acids, especially cysteine (13.38%), were found to be the most abundant after the blackening, providing insights into the Maillard reaction's impact on jujube proteins and amino acids.

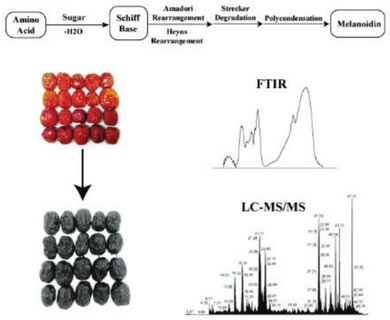

## 1 Introduction

*Ziziphus jujuba cv*. Jinsixiaozao, commonly known as jujube, belongs to the family Rhamnaceae and has been cultivated in China for centuries (Yang et al. 2021). China remains the primary producer of jujube, where it is valued both as a traditional medicinal material and a functional food (Gao et al. 2019; Zhang et al. 2020). Jujube is rich in nutrients, including vitamins, amino acids, polyphenols, flavonoids, and polysaccharides. It is recognized for its high medicinal and nutritional value and has various functional properties such as enhancing immunity, improving sleep quality, protecting gastrointestinal health, and exhibiting anti‐aging effects (Yang et al. 2021; Zhang et al. 2020). Blackened jujube is produced from red jujube through a fermentation process under controlled temperature and humidity. Its identification relies on both visual assessment and colorimetric analysis. The Maillard reaction, a non‐enzymatic browning reaction, is the key chemical process responsible for the blackening of jujube, substantially altering its appearance, aroma, and nutritional composition (Gao et al. 2019; Sun et al. 2019). During this process, sucrose content declines, while levels of fructose, glucose, and other reducing sugars increase markedly. Simultaneously, there is an increase in total phenols, 5‐hydroxymethylfurfural, organic acids, and other functional compounds. Additionally, compounds such as 2‐acetylfuran, furfural, and their derivatives contribute to a characteristic caramel flavor (Sun et al. 2019).

The Maillard reaction consists of a sequence of condensation and polymerization reactions between reducing sugars and amino acids, ultimately generating dark brown macromolecular compounds (Chen et al. 2013). The early stage involves the formation of Schiff bases through the condensation of sugars and amino acids, followed by transformations via the Amadori rearrangement, Heyns rearrangement, and Strecker degradation, leading to intermediate products such as deoxydicarbonyl sugars. In the final stage, these intermediates undergo further condensation and polymerization to yield melanoidins and nigrosine‐like substances (Ke and Li 2023). Proteins serve as essential amino group donors in the Maillard reaction. For instance, pyruvate dehydrogenase, a key enzyme linking glycolysis and the tricarboxylic acid cycle, catalyzes the conversion of pyruvate to acetyl‐CoA—a reaction that directly influences the availability of Maillard reaction precursors (Dack et al. 2017). Under Maillard reaction conditions, protein structural properties are significantly modified, which enhances their functional characteristics such as solubility, emulsifying capacity, and thermal stability (Wang et al. 2022; Zhong et al. 2019).

With growing interest in jujube, increasing research has focused on its nutritional components and health effects. As an innovative jujube product, blackened jujube is gaining recognition, and its bioactive compounds such as polysaccharides and polyphenols have attracted scholarly attention (Ke and Li 2023; Wang et al. 2022). However, due to the complex nature of the Maillard reaction in the blackening process, protein content is lower than that of other nutrients, and proteins are more difficult to isolate. Consequently, studies on jujube proteins remain limited.

This study investigates the changes in protein structure and amino acid composition during the blackening of jujube, aiming to elucidate the role of the Maillard reaction in this transformation. Ultraviolet–visible (UV–Vis) spectroscopy, Fourier‐transform infrared (FTIR) spectroscopy, and scanning electron microscopy (SEM) were used to assess protein structural properties, while amino acid analysis was employed to quantify compositional changes. These findings not only contribute to a deeper understanding of jujube's nutritional characteristics but also provide a theoretical basis for elucidating the mechanisms underlying its blackening process.

## 2 Materials and Methods

### 2.1 Materials

Ninhydrin color‐developing solution and amino acid standards were purchased from Hitachi High‐Tech Science Co. Ltd. (Osaka, Japan). Acetonitrile and methanol (HPLC grade) were obtained from Merck (Darmstadt, Germany). *Ziziphus jujuba* cv. Jinsixiaozao was provided by Guorentang Food Technology Co. Ltd. (Shandong, China). Unless otherwise specified, all other chemicals and reagents used were of analytical grade.

### 2.2 Blackening Process

The blackening process was conducted at ambient temperature (70°C ± 1°C) to simulate natural postharvest conditions. Jujube samples were placed in a climate‐controlled chamber (MIR‐254, Panasonic) with real‐time temperature monitoring using a calibrated digital thermometer (accuracy ±0.5°C). Initially, the jujubes were rehydrated for 3 h at a fruit‐to‐water ratio of 1:5. They were then removed, filter‐dried for 15 min, and sealed in bags with fresh water at a jujube‐to‐water ratio of 4:1. Blackened jujubes were obtained by heating in an oven at 65°C for 96 h.

### 2.3 Preparation of Sample of Blackened Jujube Protein

Because it is difficult to separate the protein from other jujube components, we set the starting time for the extraction of protein to 72 h. After this time, samples were taken at intervals of 6 h for protein extraction (Shevkani et al. 2015). The extraction of protein was performed according to the procedure described in a previous study with slight modifications (Kadam et al. 2017). In order to avoid protein denaturation and reduce the influence on the research content, the extraction conditions of proteins should be carefully selected (Gao et al. 2020; Zhang et al. 2009). The blackened jujube was crushed to remove impurities and degreased, and then distilled water was added to the resulting powder in the ratio of 1:10 and stirred evenly. The pH of the treated turbid liquid was adjusted to 10 with 0.05 mol/L sodium hydroxide solution (Gao et al. 2020). The turbid liquid was placed in a water bath at 50°C for 120 min and then centrifuged at 4000 rpm for 45 min. The pH of the supernatant was adjusted to 3.5 with 0.1 mol/L hydrochloric acid (Zhang et al. 2009). The excess liquid was removed by centrifugation, and the obtained protein samples were precipitated five times after being washed with water.

### 2.4 SEM Analysis of Protein Extracted From Blackened Jujube

SEM analysis was performed with slight modifications from established methods (Ma et al. 2022; Tian et al. 2023). Briefly, 10 μL of protein solution was applied to the conductive adhesive and gold‐coated for 45 s at 10 mA. A scanning electron microscope (TESCAN MIRA LMS, Shanghai, China) was used to observe sample morphology at an acceleration voltage of 3 kV using an SE2 secondary electron detector.

### 2.5 UV–Vis Spectroscopic Analysis

UV–Vis spectroscopic analysis of protein extracted from blackened jujube was conducted according to a previously described method (Tian et al. 2023). Protein extracted from blackened jujube was prepared as a 1 mg/mL solution and scanned by a UV–Vis spectrophotometer (TU‐1810PC, Beijing Purkinje General Instrument Co. Ltd., China) over the wavelength range from 200 to 700 nm.

### 2.6 FTIR Spectroscopic Analysis of Protein Structure

Protein structure was evaluated by FTIR spectroscopic analysis of protein extracted from blackened jujube. FTIR spectroscopy was used to analyze the protein extracted from blackened jujube (Li et al. 2019; Ma et al. 2022; Shevkani et al. 2015). Two‐milligram samples of blackened jujube protein were pressed into tablets with KBr and scanned over the wavenumber range of 4000–400 cm^−1^.

### 2.7 Determination of Amino Acids in Protein Extract

Amino acid content was measured according to established protocols with slight modifications (Herman et al. 2010; Tan et al. 2021). Lyophilized protein samples were hydrolyzed in 6 mol/L HCl at 110°C for 22 h. After cooling, the hydrolysate was diluted to 50 mL. One milliliter of the solution was evaporated to dryness twice and redissolved in 1 mL of sodium citrate buffer (pH 2.2). The solution was filtered through a 0.22 μm membrane and analyzed using an automatic amino acid analyzer (Hitachi LA8080, Tokyo, Japan).

### 2.8 LC–MS/MS Analysis of Protein

Liquid chromatography–tandem mass spectrometry (LC–MS/MS) was conducted based on modified methods from previous studies (Shi et al. 2018). Protein samples were fully dissolved in 100 mL of 100 mM triethylammonium bicarbonate. Trypsin was added to give an enzyme: protein ratio of 1:50 (m/m), and the samples were enzymolyzed at 37°C overnight. The samples were purified by the hydrophilic–lipophilic balance and mixed‐mode cation exchange methods. The purified samples were injected into a chromatograph, and elution was performed on a C18 column (75 μm × 25 cm, Thermo, Massachusetts, USA). The elution gradient was 5%–100% buffer B (80% acetonitrile, 20% water, 0.1% formic acid), the flow rate was 0.3 μL/min, and the elution time was 60 min. The eluate was electrically dissociated and then subjected to quantitative tandem mass spectrometry (Orbitrap Exploris 240, Thermo, Massachusetts, USA). Mass spectra were obtained in the mass range of 350–1500 m/z, with a primary mass spectrometry resolution of 60,000 (automatic gain control [AGC] target: 3e6) and a secondary mass spectrometry resolution of 15,000 (AGC target: 5e4).

### 2.9 Statistical Analysis

All experiments were performed in triplicate. Statistical analyses were conducted using SPSS 18.0 software. Student's *t*‐test and one‐way ANOVA were used to assess significance, with *p* < 0.05 considered statistically significant. OriginPro 8.5 software was used for data visualization.

## 3 Results and Discussion

### 3.1 SEM Analysis of Protein Extracted From Blackened Jujube

**FIGURE 1:**
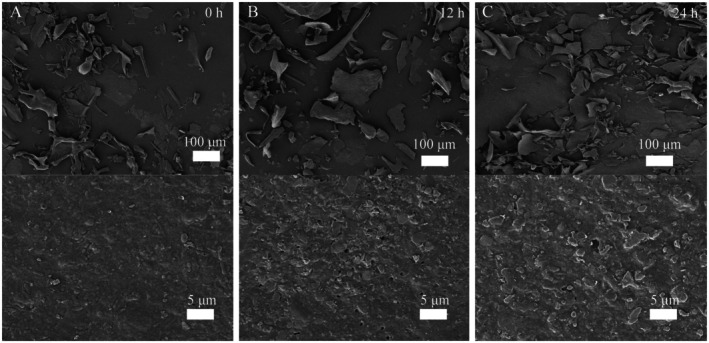
Scanning electron micrographs of protein from blackened jujube recorded at (A) 0, (B) 12, and (C) 24 h.

Scanning electron micrographs of proteins extracted from blackened jujube at various stages of blackening are presented in Figure 1. The micrographs reveal that the protein surfaces exhibited complex spatial architectures, primarily consisting of layered structures that appeared rough and irregular. As the blackening process progressed, the number of these layered structures increased. Their volume initially expanded and then decreased, while their distribution became progressively more concentrated. These structural changes are likely attributable to the Maillard reaction occurring during the blackening of jujube. This reaction disrupts covalent bonds within the proteins and reduces protein molecule aggregation, leading to a decline in the prevalence of larger layered structures and a corresponding increase in smaller layered formations. (Dursun Capar and Yalcin 2021; Tian et al. 2023).

### 3.2 UV–Vis Spectroscopic Analysis of Protein Extracted From Blackened Jujube

UV–Vis spectroscopy is a widely used analytical technique for detecting conformational changes in proteins. Protein molecules in solution exhibit UV absorption spectra due to the light absorption by side chain groups and peptide bonds between amino acid residues, and these spectra vary with alterations in protein structure. Peptide bonds typically show an absorption peak around 215 nm, while phenylalanine absorbs near 257 nm. Additionally, tyrosine and tryptophan exhibit characteristic absorption peaks at approximately 275 and 280 nm, respectively. (Yan et al. 2021, 2023).

**FIGURE 2:**
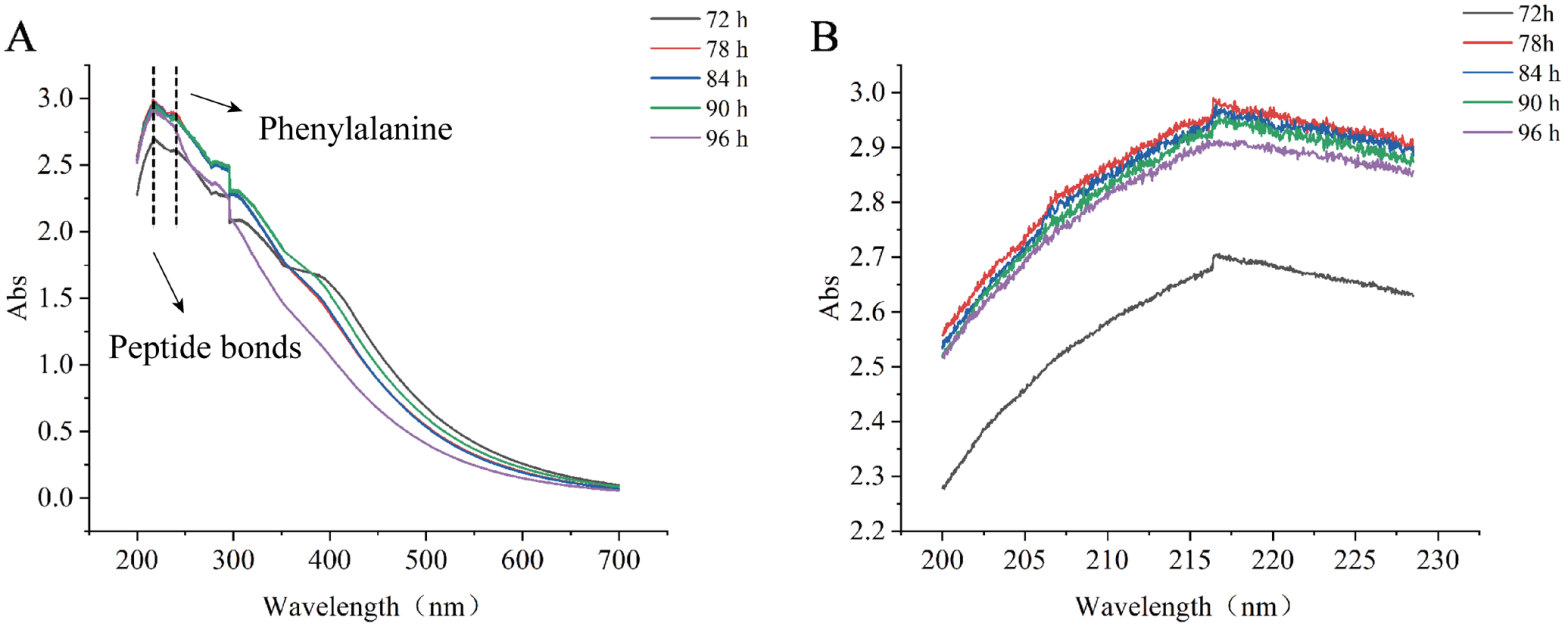
Ultraviolet‐visible spectra of protein recorded at various times during the jujube blackening process.

As shown in Figure 2, the UV absorption spectra of blackened jujube protein samples at different blackening durations all exhibited distinct absorption peaks around 215 nm. This indicates that UV light absorption by peptide bonds following protein cleavage was reflected in the spectra. Furthermore, as the blackening duration increased, the UV absorption intensity of the protein samples initially rose and then declined, reaching its maximum at 78 h. The prominent absorption near 215 nm is likely attributable to the cleavage of numerous amide bonds during the protein extraction process via alkali‐soluble acid precipitation (Zhong et al. 2019). The peak UV absorption at 78 h likely corresponds to a phase of intensified reaction between 72 and 78 h, during which surface hydrophobicity increases and aggregation‐induced burial of chromophores occurs. This reduces structural stability and results in greater exposure of peptide bonds. After 78 h, the reaction intensity diminishes, leading to a reduction in exposed peptide bonds and a subsequent decline in UV absorption.

### 3.3 FTIR Spectroscopic Analysis of Protein Extracted From Blackened Jujube

**FIGURE 3:**
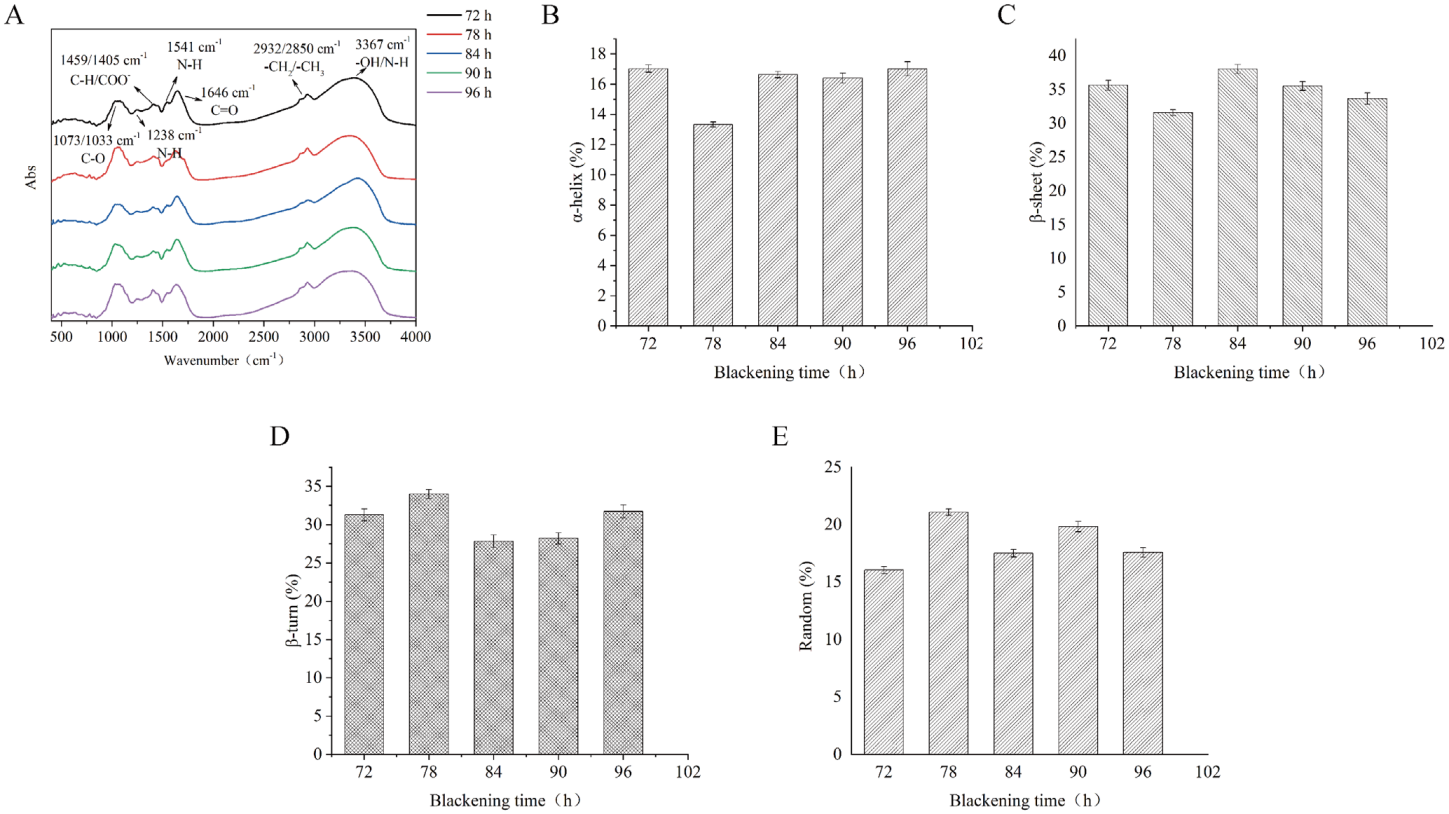
Fourier transform infrared (FTIR) spectroscopic analysis and secondary structure of blackened jujube protein at various times during the blackening process: (A) FTIR spectra, (B) β‐turn, (C) α‐helix, (D) β‐sheet, and (E) random coil.

FTIR spectroscopy is an effective technique for analyzing the secondary structure of proteins, as structural changes are reflected in the infrared spectra. The results are presented in Figure 3, Figures S1 and S2. The absorption peak at 3367 cm^−1^ lies in the amide A region and represents a combination of hydroxyl and N—H stretching vibrations. This peak is broadened due to the influence of intermolecular hydrogen bonding (Mir et al. 2023). Peaks at 2932 and 2850 cm^−1^, with zigzag shapes, are attributed to the symmetric and asymmetric stretching vibrations of methyl and methylene groups in protein side chains (Mir et al. 2023). The absorption at 1646 cm^−1^ corresponds to the amide I region and represents C=O stretching in protein amide bonds (—NH—CO—) (Yan et al. 2023), while the 1541 cm^−1^ peak in the amide II region is mainly due to N—H bending and C—N stretching in primary and secondary amines (Dursun Capar and Yalcin 2021; Wang et al. 2013). The 1456 cm^−1^ absorption is primarily associated with C—H stretching in methylene groups, while the 1405 cm^−1^ peak may correspond to symmetric C=O stretching in carboxyl groups or C—N stretching in aromatic amino groups (Zhong et al. 2019). The amide III region, represented by the 1238 cm^−1^ peak, is related to N—H and C—N stretching vibrations. Peaks at 1073 and 1033 cm^−1^ originate mainly from C—O stretching in polysaccharides and S—O bonds in proteins. The remaining peaks in the fingerprint region below 1000 cm^−1^ are attributed to various low‐frequency vibrations such as twisting and wagging motions of multiple chemical bonds (Wang et al. 2013). As shown in Figure 3A and Figure S1, the absorption peaks at 1646 and 1405 cm^−1^ changed significantly with increasing blackening duration. These peaks are primarily related to carbonyl groups in polysaccharides and amide groups in proteins, and their changes suggest the occurrence of Maillard reactions between carbonyl and free amino groups in the proteins (Yan et al. 2023; Zhong et al. 2019). In addition, the notable change near 3250 cm^−1^ indicates alterations in the hydrogen bonding system, which may have significantly influenced the protein's secondary structure.

Hydrogen bonding plays a crucial role in maintaining the secondary structure of proteins (Li et al. 2019), and differences in these bonds are reflected in regions of the IR spectrum sensitive to hydrogen interactions, particularly the amide I region. Changes in peak position and full width at half‐maximum in this region can be used to identify and quantify secondary structure elements (Mir et al. 2023; S. Yan et al. 2021). In this study, the amide I region (1600–1700 cm^−1^) was selected for peak fitting, enabling a more accurate assessment of secondary structure composition based on changes in subpeak intensity and location (Yang et al. 2019). The fitting results revealed that the relative content of α‐helix structures ranged from 13% to 18%, β‐sheet and β‐turn structures together accounted for approximately 30%, and random coil structures ranged from 16% to 21% (Figure 3B–3E). The content of α‐helix structures decreased from 17.03% to 13.33%, and both α‐helix and β‐sheet contents dropped significantly between 72 and 78 h (*p* < 0.001). These changes are consistent with Maillard reaction–induced protein crosslinking, where advanced glycation end products (AGEs) derived from lysine or arginine residues disrupt native protein folding (Mir et al. 2023). This phenomenon may be explained by structural modifications in proteins due to covalent bond rearrangements occurring during the Maillard reaction between 72 and 78 h. These changes result in a more stable protein structure by 78 h, at which point the reaction intensity decreases and α‐helix and β‐sheet contents stabilize. As protein structure changes, protein functionality and surface hydrophilicity are also likely affected (Mir et al. 2023; Yang et al. 2019). These findings align with the UV–Vis spectroscopy results and further confirm that proteins actively participate in chemical transformations—primarily the Maillard reaction—during the jujube blackening process.

### 3.4 Amino Acid Analysis of Protein Extracted From Blackened Jujube

**FIGURE 4:**
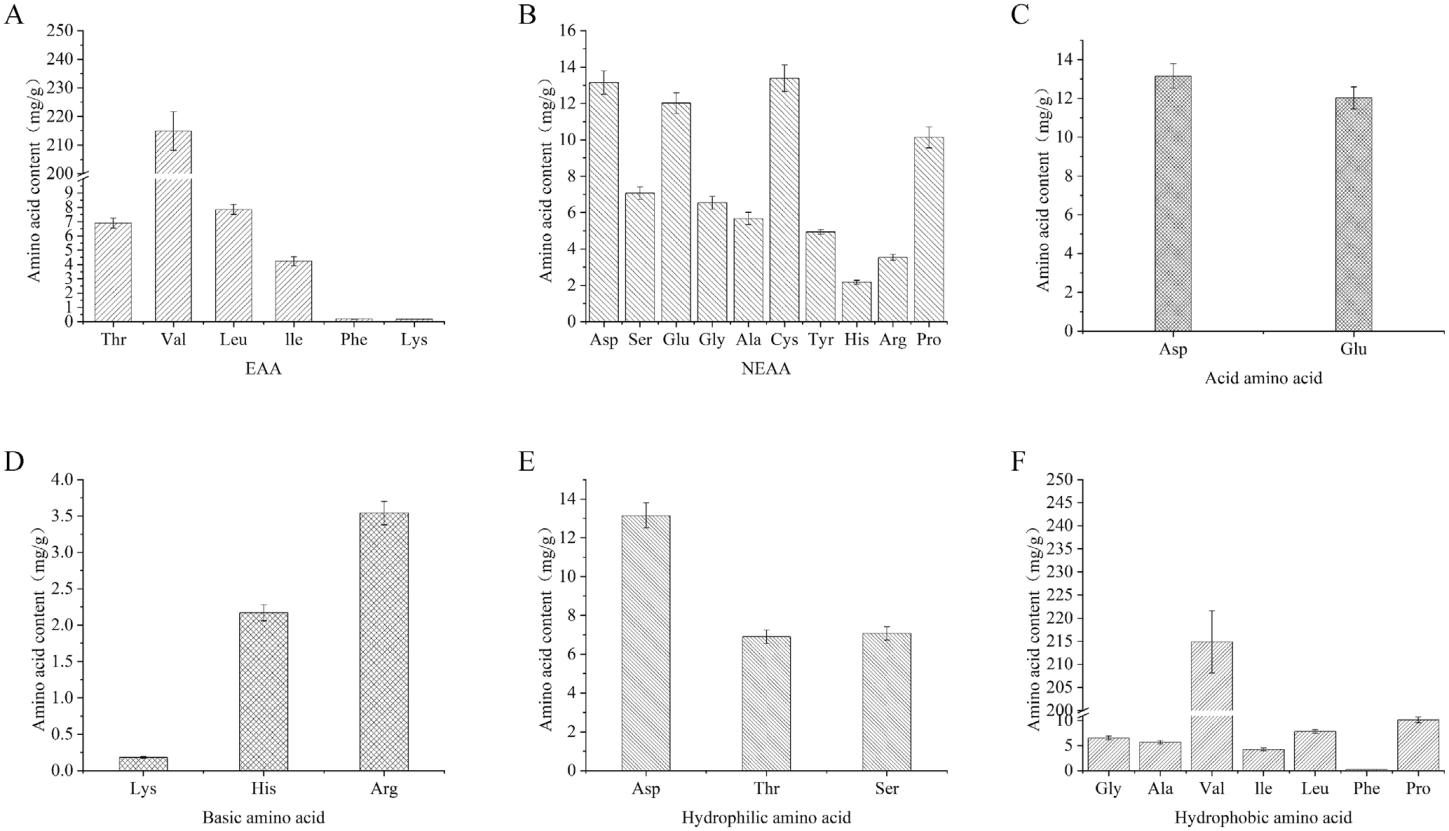
Amino acid analysis of protein extracted from blackened jujube: (A) essential amino acids, (B) nonessential amino acids, (C) acidic amino acids, (D) basic amino acids, (E) hydrophilic amino acids, and (F) hydrophobic amino acids.

Building on the preceding analyses, blackened jujube protein samples from the 78‐h time point were selected for further investigation, including amino acid profiling. As shown in Figure 4, a total of 17 amino acids were detected, comprising 7 essential and 10 nonessential amino acids. Essential amino acids accounted for 74.9% of the total amino acid content, approximately three times the content of nonessential amino acids. Figure 4A shows that among the essential amino acids, valine (Val) had a markedly higher concentration than the others, representing 68.69% of the total amino acid content. In contrast, Figure 4B indicates no significant differences among the concentrations of nonessential amino acids. Cysteine (Cys) had the highest content among them, accounting for 13.38% of the total amino acid content, with aspartic acid (Asp) and glutamic acid (Glu) exhibiting similar levels. A comparison of Figure 4A,B reveals that Val is the predominant amino acid in jujube protein, followed by Cys, Asp, and Glu. Val, classified as both a branched‐chain and glucogenic amino acid, plays key physiological roles in promoting growth, tissue repair, and blood glucose regulation (Gao et al. 2018; Herman et al. 2010). Although Cys, Asp, and Glu are nonessential, all exhibit strong antioxidant properties, contributing to the antioxidant capacity of jujube proteins (Kadam et al. 2017).

As illustrated in Figure 4C,D, two acidic amino acids were identified in the sample, accounting for 8.05% of the total amino acid content, while three basic amino acids accounted for only 1.88%. This imbalance suggests that jujube protein has a relatively high acidic amino acid content, reinforcing the acidic properties of the protein.

Figure 4E,F reveal the presence of seven hydrophobic amino acids, comprising 79.75% of the total amino acid content, with Val being the most abundant. In contrast, three hydrophilic amino acids were detected, comprising 8.67% of the total, with Asp showing the highest concentration. Hydrophobic amino acids tend to repel water from hydrophobic regions and encourage hydrogen bond formation between amide groups, leading to decreased protein solubility in aqueous environments (Deng et al. 2019; Gao et al. 2018). Further analysis indicated that the content of hydrophobic amino acids at 78 h was higher than at most other time points. A high concentration of hydrophobic amino acids may enhance protein stability by facilitating the formation of a compact inner core. This observation aligns with findings in roasted coffee, where hydrophobic amino acids play a dominant role in the formation of Maillard‐derived flavors (Casal et al. 2005). Consequently, the protein structure in jujube at 78 h appears relatively stable, consistent with results from FTIR and UV–Vis spectroscopy (Deng et al. 2019). The post‐blackening increase in hydrophobic amino acids also corresponds with the formation of melanoidins. To enhance antioxidant capacity while minimizing nutrient loss—particularly lysine depletion—low‐temperature processing protocols (e.g., 50°C–60°C) could be adopted. This suggests that optimizing blackening duration could strike a balance between flavor development via the Maillard reaction and nutrient preservation. For example, terminating the blackening process at 72 h may retain a higher level of essential amino acids while still achieving desirable browning and flavor characteristics.

### 3.5 Liquid Chromatography–Tandem Mass Spectrometry Analysis of Protein Extracted From Blackened Jujube

Blackened jujube protein at 78 h was enzymatically hydrolyzed using trypsin and analyzed by liquid chromatography–tandem mass spectrometry (LC–MS/MS). The resulting data are presented in Table 1 and Figure S3. Through further analysis and screening, a total of 33 confidently identified proteins were obtained. These included enzymes, membrane skeleton proteins, transporters, antigens, antibodies, ion channels, and other protein types. Among these, enzymes constituted the largest proportion. Molecular weight analysis revealed that blackened jujube protein contains proteins with a range of molecular weights, with the most abundant group exhibiting molecular weights around 60 kDa.

**TABLE 1:** Protein components of blackened jujube protein determined by liquid chromatography–tandem mass spectrometry.

Identified protein name	Score	Coverage (%)	Unique	Avg. Mass
Pectinesterase	185.31	26	13	66,005
L‐ascorbate oxidase	309.93	63	40	60,483
(R)‐mandelonitrile lyase (pyruvate dehydrogenase)	275.92	48	9	60,324
Galactose oxidase	220.88	44	27	59,917
Probable glucan 1 3‐beta‐glucosidase A isoform X2	153.26	18	8	56,305
Low‐temperature‐induced protein	186.2	26	9	51,532
Probable polygalacturonase	152.62	22	9	49,223
Aspartyl protease AED3	133.43	19	9	46,575
Basic 7S globulin	284.89	58	51	46,065
GDSL esterase/lipase	238.42	51	4	40,883
DNA damage‐repair/toleration protein	207.89	51	19	39,649
Glyceraldehyde‐3‐phosphate dehydrogenase	138.42	24	3	36,904
Xyloglucan endotransglucosylase/hydrolase	147.58	26	8	34,116
Xyloglucan endotransglucosylase/hydrolase	136.99	22	7	33,314
23 kDa jasmonate‐induced protein	100.78	22	6	26,593
Glycine‐rich cell wall structural protein	132.23	22	4	26,380
Thaumatin‐like protein	294.4	52	3	25,505

Predicting a protein's spatial (three‐dimensional) structure based on its primary amino acid sequence remains a key focus in structural biology. If the sequence identity between two proteins exceeds 30%, their folding processes and spatial structures are generally considered to be similar (Mosalaganti et al. 2022). Comparative analysis showed that the most abundant proteins in blackened jujube shared structural and functional similarities with L‐ascorbate oxidase, pyruvate dehydrogenase, and galactose oxidase. The presence of L‐ascorbate oxidase and galactose oxidase supports the conclusion that jujube protein possesses strong antioxidant activity, consistent with findings from the amino acid analysis. Numerous studies have demonstrated that the antioxidant activity of proteins increases significantly following the Maillard reaction (Tan et al. 2021; Wang et al. 2013). Pyruvate dehydrogenase plays a key role in the oxidative decarboxylation of pyruvate, facilitating the synthesis of amino acid derivatives such as alanine, valine, and leucine. The observed reduction in amino acid content after jujube blackening further supports the occurrence of the Maillard reaction. These findings highlight the reaction's role in modifying amino donors during the blackening process (Mosalaganti et al. 2022; Shi et al. 2018). Moreover, the aggregation of pyruvate dehydrogenase (~60 kDa) and the associated decline in α‐helix content suggest that structural changes may hinder enzymatic hydrolysis. Aggregates enriched in β‐sheet structures are known to resist proteolytic cleavage due to steric hindrance (Xu et al. 2020). Additionally, Maillard reaction‐derived crosslinks—such as advanced glycation end products formed from lysine and arginine—may further reduce digestibility by obstructing enzyme access to cleavage sites (Tarley et al. 2021).

### 3.6 Amino Acid Analysis of Jujube During the Blackening Process

**FIGURE 5:**
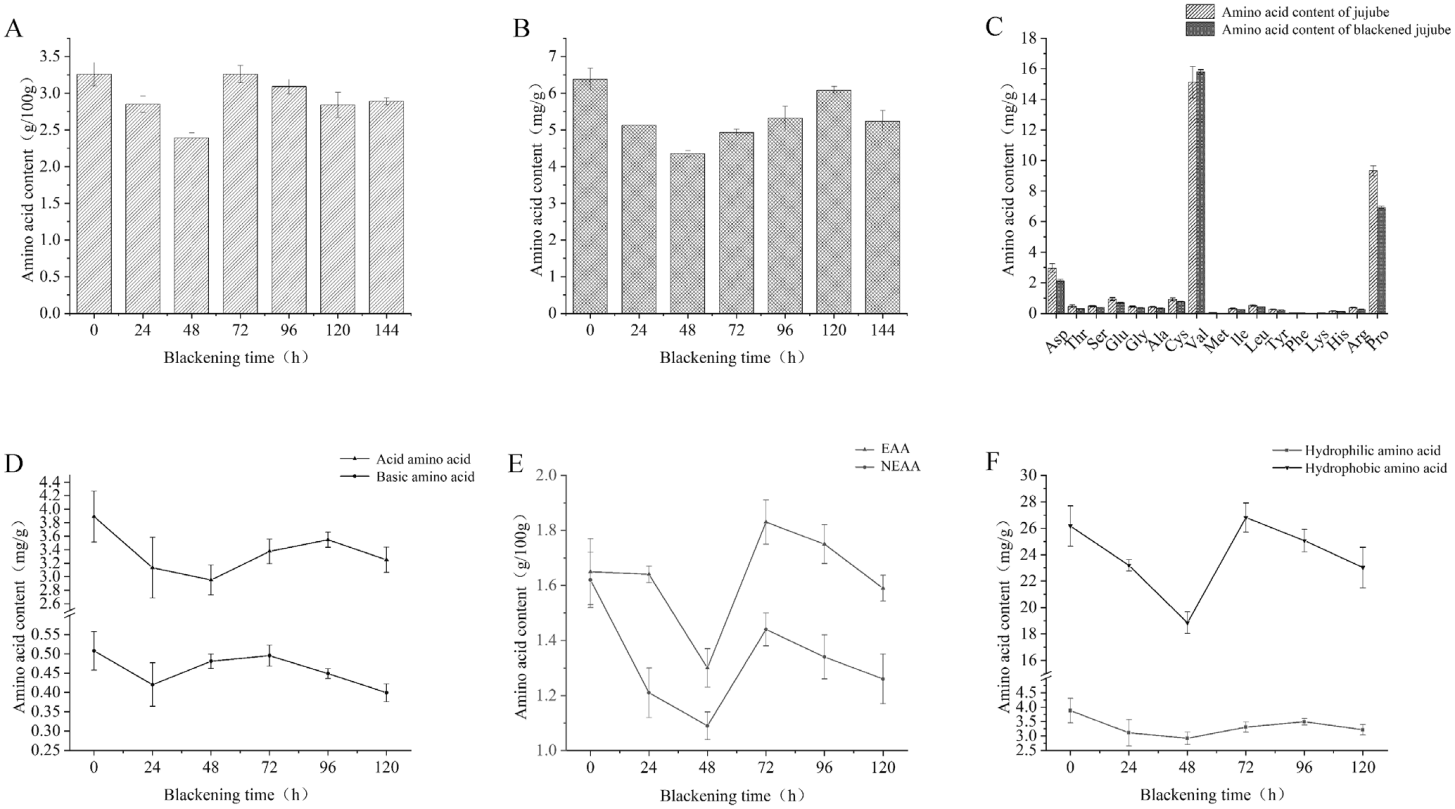
Varieties and contents of different amino acids among total amino acids and free amino acids in jujube during blackening: (A) total amino acids, (B) free amino acids, (C) comparison of single amino acids, (D) changes in essential amino acids and nonessential amino acids, (E) changes in acidic amino acids and basic amino acids, and (F) changes in hydrophilic amino acids and hydrophobic amino acids.

As shown in Figure 5A,B, both total and free amino acid contents exhibited a gradual and consistent trend throughout the blackening process. A comparison of the two figures indicates a partial correlation between total and free amino acid levels. This pattern may be attributed to the complex chemical reactions occurring during the jujube blackening process. In addition to the nonenzymatic browning reactions dominated by the Maillard reaction, protein hydrolysis also significantly influenced amino acid content. Free amino acids, serving as amino donors, are consumed in the Maillard reaction through interactions with reducing sugars. This explains the significant 31.5% reduction in free amino acid content during the early phase of blackening (0–48 h) (*p* < 0.05). Both total and free amino acid contents decreased significantly over time, confirming that amino acids were actively consumed in the Maillard reaction (Tan et al. 2021; Zhong et al. 2019).

According to Figure 5C, 17 amino acids were detected in blackened jujube protein, including seven essential amino acids. Among these, valine, proline, and aspartic acid were the most abundant, with contents of 1.53, 0.67, and 0.21 g/100 g, respectively, accounting for 68.69%, 30.08%, and 9.43% of total amino acids. Methionine, phenylalanine, lysine, and histidine contents showed no significant changes (*p* > 0.05) across most time points. This may be due to their lower reactivity in Maillard reactions, possibly linked to steric hindrance. For example, valine's branched‐chain structure may restrict participation in Schiff base formation due to limited accessibility of its ε‐amino group (Chen et al. 2025). The dominance of valine may enhance the nutritional profile of blackened jujube, as it plays a key role in branched‐chain amino acid metabolism, supporting muscle protein synthesis and glycemic control.

Notably, phenylalanine levels remained unchanged during blackening, while most other amino acids showed marked reductions. Proline and aspartic acid contents decreased by 0.24 (*F* (12, 26) = 5.52, *p* < 0.001) and 0.09 g/100 g (*F* (12, 26) = 15.27, *p* < 0.001), respectively, while other amino acids declined by similar amounts. These findings indicate that proline and aspartic acid were the primary amino acids involved in the Maillard reaction. The observed increase in valine content may reflect exposure of unreacted amino groups that offset reductions caused by covalent modification (Chen et al. 2013).

Figure 5D shows that the content trends of essential and nonessential amino acids during the blackening process were largely similar, indicating that both types were involved in the Maillard reaction. However, by comparing their absolute levels and changes, it is evident that nonessential amino acids were the primary contributors. Figure 5E shows that acidic and basic amino acid contents were comparable across blackening stages, suggesting that both classes played important roles in Maillard‐related reactions.

Figure 5F highlights that the content of hydrophobic amino acids was substantially higher than that of hydrophilic amino acids, and their fluctuation during blackening was more pronounced. This implies that hydrophobic amino acids were the main contributors to the Maillard reaction. A comprehensive analysis of Figure 5 indicates that the trends in amino acid content across different classifications were generally consistent and exhibited fluctuating behavior. This phenomenon may result from the degradation of intermediate products and ongoing Amadori rearrangements, which can influence both amino acid conformation and the reactivity of free amino groups with other molecular structures (Chen et al. 2013; Zha et al. 2021; Zhong et al. 2019).

## 4 Conclusions

This study investigated the alterations in proteins and amino acids during the jujube blackening process. As the duration of blackening increased, the microstructure of jujube protein underwent significant changes, including an increase in the number of layered structures and a reduction in their volume. FTIR and UV spectroscopic analyses revealed marked transitions in the secondary structures of jujube proteins throughout the process, with the most substantial changes observed at 78 h. Upon darkening, valine emerged as the most abundant essential amino acid, accounting for 68.69% of the total amino acid content, while cysteine was the most prevalent nonessential amino acid at 13.38%. The predominant protein identified had a molecular weight of approximately 60 kDa. Notably, the contents of α‐helix and β‐sheet structures decreased significantly, whereas β‐turn structures increased. Proteins from the 78‐h time point were selected for further characterization. The content of acidic amino acids exceeded that of basic amino acids, contributing to the acidic profile of blackened jujube. Additionally, the higher proportion of hydrophobic amino acids relative to hydrophilic ones enhanced protein stability and antioxidant capacity. LC–MS/MS analysis identified pyruvate dehydrogenase and several antioxidant enzymes as major protein components, corroborating the amino acid findings and confirming the involvement of the Maillard reaction in modifying amino donors. Throughout the blackening process, both total and free amino acid levels fluctuated significantly, with most amino acids showing substantial decreases, except for a few exceptions. These fluctuations reflect the consumption and transformation of amino acids via chemical reactions, particularly the Maillard reaction. This study not only deepens the current understanding of the nutritional properties of jujube but also elucidates the role of protein and amino acid transformations during the melanization process. The findings strongly suggest that the blackening process is closely related to Maillard‐type reactions and provide a theoretical foundation for future research into the mechanisms underlying jujube blackening.

## Author Contributions

**Weihao Kong:** conceptualization, experiments, software, data curation, writing – original draft. **Xin Sun:** investigation, visualization, software, writing – review and editing. **Xin Zhang:** writing – review and editing. **Yuxiao Wang:** investigation, visualization. **Yifei Zhao:** conceptualization, review and editing. **Lin Gao:** investigation, visualization. **Lingwei Bu:** experiments, software. supervision, project administration. **Rentang Zhang:** conceptualization, methodology, supervision, funding acquisition, writing – review and editing.

## Conflicts of Interest

The authors declare no conflicts of interest.

## Supporting information


Appendix S1.

## Data Availability

The data that support the findings of this study are available on request from the corresponding author. The data are not publicly available due to privacy or ethical restrictions.
