# Supplementary material for: Proteomic and Amino Acid Dynamics During Jujube Blackening: Structural Transformations and Maillard Reaction Linkages
Source: Food Sci Nutr. 2025 Jul 25;13(7):e70644. doi: 10.1002/fsn3.70644 (PMC12290483; doi:10.1002/fsn3.70644)
Supplement: Supplementary file 1 — Appendix S1. [file FSN3-13-e70644-s001.doc]

**Proteomic and Amino Acid Dynamics During Jujube Blackening: Structural Transformations and Maillard Reaction Linkages**

Weihao Kong^a,1^, Xin Sun^a,1^, Xin Zhang^a^, Yuxiao Wang^a^, Yifei Zhao^a^, Lin Gao^a^, Lingwei Bu^b^, Rentang Zhanga,b[[1]](#footnote-2)*

^a^ College of Food Science and Engineering, Shandong Agricultural University, Tai'an, Shandong, 271018, China

^b^ Laoling Healthy Food Industry Technology Research Institute, Dezhou, 253600, China

*1 Definition of Blackening Stage*

The blackening status of jujube was determined through visual observation and quantitative chemical. Samples showing homogeneous dark brown/black pigmentation under controlled lighting (D65 standard) were categorized as “blackened”. The Chemical thresholds was defined as 5-HMF ≥ 0.1 mg/g (HPLC detection, Shimadzu LC-20A), and Melanoidins absorbance ≥ 0.8 at 420 nm (UV-Vis spectrophotometer, Shimadzu UV-2600i).

**Table S1** Amino acid analysis of blackened jujube at different durations.

| Amino acid | Amino acid content (g/100g) | | | | | | | | | | | | |
| --- | --- | --- | --- | --- | --- | --- | --- | --- | --- | --- | --- | --- | --- |
|  | 0 h | 12 h | 24 h | 36 h | 48 h | 60 h | 72 h | 84 h | 96 h | 108 h | 120 h | 132 h | 144 h |
| Asp | 0.30±0.03 | 0.23±0.02 | 0.24±0.03 | 0.30±0.04 | 0.23±0.02 | 0.22±0.03 | 0.25±0.01 | 0.22±0.02 | 0.27±0.01 | 0.30±0.03 | 0.25±0.01 | 0.26±0.02 | 0.21±0.01 |
| Thr | 0.04±0.01 | 0.03±0.00 | 0.03±0.01 | 0.04±0.01 | 0.03±0.00 | 0.04±0.01 | 0.04±0.00 | 0.03±0.00 | 0.03±0.00f | 0.04±0.00 | 0.03±0.00 | 0.04±0.00 | 0.03±0.00 |
| Ser | 0.05±0.00 | 0.04±0.00 | 0.04±0.01 | 0.05±0.01 | 0.03±0.00 | 0.04±0.01 | 0.04±0.00 | 0.03±0.00 | 0.04±0.00 | 0.05±0.00 | 0.04±0.00 | 0.04±0.00 | 0.04±0.00 |
| Glu | 0.09±0.01 | 0.08±0.01 | 0.08±0.01 | 0.09±0.01 | 0.07±0.00 | 0.08±0.01 | 0.09±0.00 | 0.07±0.01 | 0.08±0.00 | 0.09±0.01 | 0.08±0.00 | 0.08±0.01 | 0.07±0.00 |
| Gly | 0.04±0.00 | 0.04±0.00 | 0.04±0.01 | 0.04±0.01 | 0.03±0.00 | 0.04±0.01 | 0.04±0.00 | 0.03±0.00 | 0.04±0.00 | 0.04±0.00 | 0.04±0.00 | 0.04±0.00 | 0.04±0.00 |
| Ala | 0.04±0.00 | 0.03±0.00 | 0.04±0.00 | 0.04±0.01 | 0.03±0.00 | 0.04±0.01 | 0.04±0.00 | 0.03±0.00 | 0.04±0.00 | 0.04±0.00 | 0.04±0.00 | 0.04±0.00 | 0.03±0.00 |
| Cys | 0.09±0.01 | 0.08±0.00 | 0.08±0.01 | 0.09±0.01 | 0.07±0.00 | 0.09±0.01 | 0.09±0.00 | 0.07±0.01 | 0.09±0.00 | 0.09±0.01 | 0.08±0.00 | 0.09±0.01 | 0.08±0.00 |
| Val | 1.51±0.15 | 1.29±0.11 | 1.55±0.19 | 1.49±0.18 | 1.19±0.05 | 1.60±0.28 | 1.69±0.08 | 1.47±0.17 | 1.63±0.05 | 1.75±0.16 | 1.47±0.09 | 1.72±0.15 | 1.58±0.07 |
| Met | 0.00±0.00 | 0.00±0.00 | 0.00±0.00 | 0.01±0.00 | 0.01±0.00 | 0.00±0.00 | 0.01±0.00 | 0.00±0.00 | 0.01±0.00 | 0.01±0.00 | 0.01±0.00 | 0.01±0.00 | 0.00±0.00 |
| lle | 0.03±0.00 | 0.03±0.00 | 0.00±0.00 | 0.03±0.00 | 0.02±0.00 | 0.03±0.00 | 0.03±0.00 | 0.02±0.00 | 0.03±0.00 | 0.03±0.00 | 0.03±0.00 | 0.03±0.00 | 0.02±0.00 |
| Leu | 0.05±0.01 | 0.04±0.00 | 0.05±0.01 | 0.05±0.01 | 0.04±0.00 | 0.05±0.01 | 0.05±0.00 | 0.04±0.00 | 0.05±0.00 | 0.05±0.00 | 0.04±0.00 | 0.05±0.00 | 0.04±0.00 |
| Tyr | 0.02±0.00 | 0.02±0.00 | 0.02±0.00 | 0.02±0.00 | 0.02±0.00 | 0.02±0.00 | 0.02±0.00 | 0.02±0.00 | 0.02±0.00 | 0.02±0.00 | 0.02±0.00 | 0.02±0.00 | 0.02±0.00 |
| Phe | 0.00±0.00 | 0.00±0.00 | 0.00±0.00 | 0.00±0.00 | 0.00±0.00 | 0.00±0.00 | 0.00±0.00 | 0.00±0.00 | 0.00±0.00 | 0.00±0.00 | 0.00±0.00 | 0.00±0.00 | 0.00±0.00 |
| Lys | 0.00±0.00 | 0.01±0.00 | 0.00±0.00 | 0.00±0.00 | 0.01±0.00 | 0.00±0.00 | 0.01±0.00 | 0.01±0.00 | 0.00±0.00 | 0.00±0.00 | 0.00±0.00 | 0.00±0.00 | 0.00±0.00 |
| His | 0.01±0.00 | 0.01±0.00 | 0.01±0.00 | 0.01±0.00 | 0.01±0.00 | 0.01±0.00 | 0.01±0.00 | 0.01±0.00 | 0.01±0.00 | 0.01±0.00 | 0.01±0.00 | 0.01±0.00 | 0.01±0.00 |
| Arg | 0.04±0.00 | 0.03±0.00 | 0.03±0.00 | 0.00±0.00 | 0.03±0.00 | 0.03±0.01 | 0.03±0.00 | 0.03±0.00 | 0.03±0.00 | 0.03±0.00 | 0.03±0.00 | 0.03±0.00 | 0.02±0.00 |
| Pro | 0.93±0.08 | 0.63±0.04 | 0.64±0.08 | 0.70±0.08 | 0.55±0.05 | 0.62±0.11 | 0.82±0.04 | 0.64±0.07 | 0.72±0.02 | 0.71±0.07 | 0.68±0.04 | 0.76±0.06 | 0.69±0.03 |

**Table S2** Free amino acids analysis of blackened jujube at different durations.

| Amino acid | Amino acid content (100mg/g) | | | | | |
| --- | --- | --- | --- | --- | --- | --- |
|  | 0 h | 24 h | 48 h | 72 h | 96 h | 120 h |
| Asp | 7.84±0.37 | 5.57±0.17 | 6.14±0.12 | 8.34±0.16 | 8.79±0.54 | 8.37±0.15 |
| Thr | 59.38±2.77 | 40.91±1.22 | 35.82±0.71 | 35.57±0.67 | 38.98±2.41 | 28.68±0.51 |
| Ser | 0.54±0.02 | 3.37±0.09 | 3.86±0.08 | 3.69±0.07 | 3.49±0.22 | 3.10±0.05 |
| Glu | 5.97±0.28 | 3.24±0.10 | 2.74±0.06 | 4.01±0.08 | 2.38±0.15 | 2.28±0.04 |
| Gly | 1.84±0.09 | 1.25±0.04 | 1.34±0.03 | 0.02±0.00 | 1.46±0.09 | 0.04±0.00 |
| Ala | 0.30±0.02 | 0.33±0.01 | 2.13±0.04 | 2.13±0.05 | 2.16±0.13 | 2.72±0.05 |
| Cys | 3.41±0.16 | 2.74±0.08 | 4.40±0.09 | 4.47±0.09 | 4.39±0.27 | 4.54±0.08 |
| Val | 0.00±0.00 | 6.51±0.19 | 21.34±0.42 | 11.64±0.22 | 0.00±0.00 | 75.18±1.35 |
| Met | 0.00±0.00 | 0.00±0,00 | 0.00±0.00 | 0.02±0.00 | 0.11±0.03 | 0.03±0.00 |
| lle | 0.04±0.00 | 0.00±0,00 | 0.04±0.00 | 0.16±0.00 | 0.16±0.01 | 0.00±0.00 |
| Leu | 2.22±0.10 | 2.21±0.07 | 2.71±0.05 | 2.87±0.05 | 1.52±0.10 | 1.59±0.03 |
| Tyr | 6.88±0.32 | 6.07±0.18 | 5.86±0.12 | 5.29±0.10 | 4.69±0.29 | 6.45±0.11 |
| Phe | 1.93±0.09 | 1.90±0.06 | 2.03±0.04 | 2.14±0.04 | 1.73±0.11 | 2.72±0.05 |
| Lys | 0.46±0.02 | 0.66±0.02 | 1.17±0.02 | 0.11±0.00 | 1.09±0.07 | 5.82±0.10 |
| His | 0.10±0.00 | 0.02±0.00 | 0.32±0.03 | 0.29±0.00 | 0.44±0.03 | 0.56±0.01 |
| Arg | 3.17±0.15 | 1.37±0.04 | 1.02±0.02 | 1.36±0.03 | 1.32±0.08 | 1.63±0.03 |
| Pro | 543.91±25.37 | 436.62±13.02 | 343.49±6.84 | 410.95±7.72 | 459.35±28.56 | 464.66±8.32 |

**Table S3** **Identification of the proteins extracted from the blackened jujube with liquid chromatography–tandem mass spectrometry.**

|  | Identified Protein name | Score | Coverage (%) | Unique | Avg. Mass |
| --- | --- | --- | --- | --- | --- |
| 1 | Pectinesterase | 185.31 | 26 | 13 | 66005 |
| 2 | L-ascorbate oxidase | 309.93 | 63 | 40 | 60483 |
| 3 | (R)-mandelonitrile lyase (pyruvate dehydrogenase) | 275.92 | 48 | 9 | 60324 |
| 4 | Galactose oxidase | 220.88 | 44 | 27 | 59917 |
| 5 | Probable glucan 1 3-beta-glucosidase A isoform X2 | 153.26 | 18 | 8 | 56305 |
| 6 | Low-temperature-induced protein | 186.2 | 26 | 9 | 51532 |
| 7 | Probable polygalacturonase | 152.62 | 22 | 9 | 49223 |
| 8 | Aspartyl protease AED3 | 133.43 | 19 | 9 | 46575 |
| 9 | Basic 7S globulin | 284.89 | 58 | 51 | 46065 |
| 10 | GDSL esterase/lipase | 238.42 | 51 | 4 | 40883 |
| 11 | DNA damage-repair/toleration protein | 207.89 | 51 | 19 | 39649 |
| 12 | Glyceraldehyde-3-phosphate dehydrogenase | 138.42 | 24 | 3 | 36904 |
| 13 | Xyloglucan endotransglucosylase/hydrolase | 147.58 | 26 | 8 | 34116 |
| 14 | Xyloglucan endotransglucosylase/hydrolase | 136.99 | 22 | 7 | 33314 |
| 15 | 23 kDa jasmonate-induced protein | 100.78 | 22 | 6 | 26593 |
| 16 | Glycine-rich cell wall structural protein | 132.23 | 22 | 4 | 26380 |
| 17 | Thaumatin-like protein | 294.4 | 52 | 3 | 25505 |
| 18 | Pectinesterase | 185.31 | 26 | 13 | 66005 |
| 19 | L-ascorbate oxidase | 309.93 | 63 | 40 | 60483 |
| 20 | (R)-mandelonitrile lyase | 275.92 | 48 | 9 | 60324 |
| 21 | Galactose oxidase | 220.88 | 44 | 27 | 59917 |
| 22 | Probable glucan 1 3-beta-glucosidase A isoform X2 | 153.26 | 18 | 8 | 56305 |
| 23 | Low-temperature-induced protein | 186.2 | 26 | 9 | 51532 |
| 24 | Probable polygalacturonase | 152.62 | 22 | 9 | 49223 |
| 25 | Aspartyl protease AED3 | 133.43 | 19 | 9 | 46575 |
| 26 | Basic 7S globulin | 284.89 | 58 | 51 | 46065 |
| 27 | GDSL esterase/lipase | 238.42 | 51 | 4 | 40883 |
| 28 | DNA damage-repair/toleration protein | 207.89 | 51 | 19 | 39649 |
| 29 | Glyceraldehyde-3-phosphate dehydrogenase | 138.42 | 24 | 3 | 36904 |
| 30 | Xyloglucan endotransglucosylase/hydrolase | 147.58 | 26 | 8 | 34116 |
| 31 | Xyloglucan endotransglucosylase/hydrolase | 136.99 | 22 | 7 | 33314 |
| 32 | 23 kDa jasmonate-induced protein | 100.78 | 22 | 6 | 26593 |
| 33 | Glycine-rich cell wall structural protein | 132.23 | 22 | 4 | 26380 |
| 34 | Thaumatin-like protein | 294.4 | 52 | 3 | 25505 |


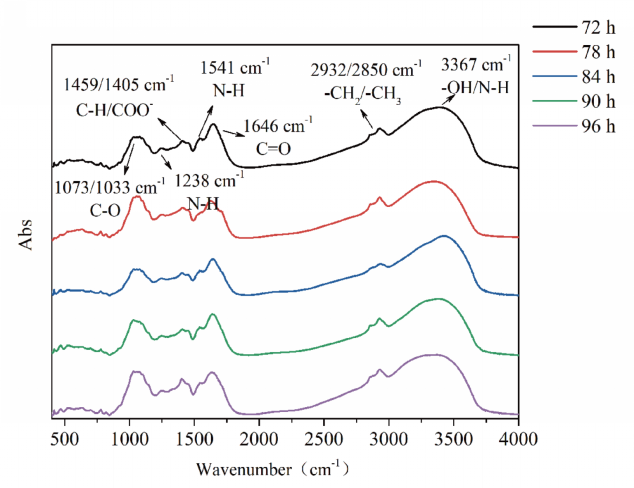


**Figure S1** Ultraviolet-visible spectra of protein recorded at 72 h, 78 h, 84 h, 90 h, and 96 h.


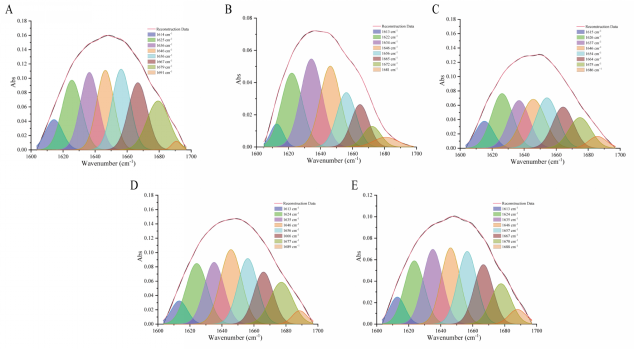


**Figure S2** Secondary structure analysis of proteins extracted from blackened jujube at 72 h (A), 78 h (B), 84 h (C), 90 h (D), and 96 h (E).


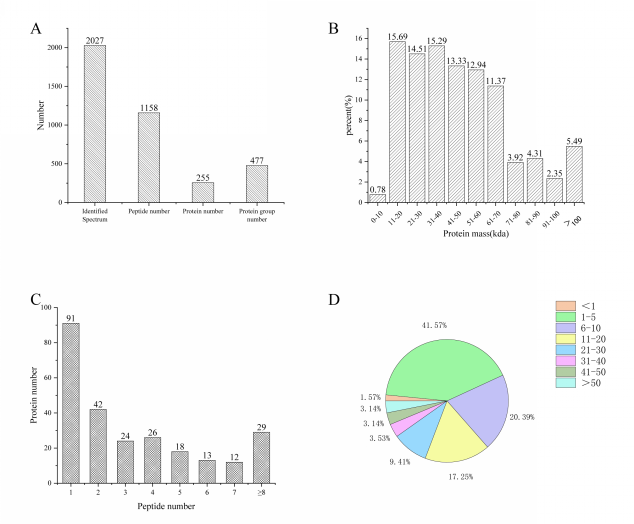


**Figure S3** Components analysis of proteins extracted from blackened jujube determined by liquid chromatography–tandem mass spectrometry: (A) the total number of proteins in blackened jujube, (B) molecular weight distribution of proteins in blackened jujube, (C) peptide number distribution of proteins, (D) distribution of different protein coverage.

1. ^*1^ The authors (W. H. Kong and X. Sun) contributed equally to this work.

   *Corresponding author. Email: rentangzhang@163.com (R. Zhang).

   The postal address: College of Food Science and Engineering, Shandong Agricultural University, Tai'an, Shandong, 271018, China [↑](#footnote-ref-2)
